# Evolution of Space Dependent Growth in the Teleost *Astyanax mexicanus*

**DOI:** 10.1371/journal.pone.0041443

**Published:** 2012-08-01

**Authors:** Natalya D. Gallo, William R. Jeffery

**Affiliations:** Department of Biology, University of Maryland, College Park, Maryland, United States of America; Laboratoire Arago, France

## Abstract

The relationship between growth rate and environmental space is an unresolved issue in teleosts. While it is known from aquaculture studies that stocking density has a negative relationship to growth, the underlying mechanisms have not been elucidated, primarily because the growth rate of populations rather than individual fish were the subject of all previous studies. Here we investigate this problem in the teleost *Astyanax mexicanus*, which consists of a sighted surface-dwelling form (surface fish) and several blind cave-dwelling (cavefish) forms. Surface fish and cavefish are distinguished by living in spatially contrasting environments and therefore are excellent models to study the effects of environmental size on growth. Multiple controlled growth experiments with individual fish raised in confined or unconfined spaces showed that environmental size has a major impact on growth rate in surface fish, a trait we have termed space dependent growth (SDG). In contrast, SDG has regressed to different degrees in the Pachón and Tinaja populations of cavefish. Mating experiments between surface and Pachón cavefish show that SDG is inherited as a dominant trait and is controlled by multiple genetic factors. Despite its regression in blind cavefish, SDG is not affected when sighted surface fish are raised in darkness, indicating that vision is not required to perceive and react to environmental space. Analysis of plasma cortisol levels showed that an elevation above basal levels occurred soon after surface fish were exposed to confined space. This initial cortisol peak was absent in Pachón cavefish, suggesting that the effects of confined space on growth may be mediated partly through a stress response. We conclude that *Astyanax* reacts to confined spaces by exhibiting SDG, which has a genetic component and shows evolutionary regression during adaptation of cavefish to confined environments.

## Introduction

Teleosts raised in confined environmental space commonly have stunted growth [1]–[4], but little is known about the underlying mechanisms. This gap in our knowledge of the effects of the local environment on teleost growth is primarily due to the focus of previous studies on multiple individuals, making it impossible to distinguish between the relative roles of environmental size and social interactions between fish [5], [6]. When seen, decreased or stunted growth in small aquaria or at high stocking densities is frequently attributed to the gradual decline of environmental factors, such as water quality, oxygen and food availability, the buildup of excretions, or limited exercise opportunity. This leads to the perception that the reduced growth rate of teleosts in confined space is determined solely by extrinsic environmental factors.

An alternative explanation for the effects of confined space on growth is that teleosts may have evolved an intrinsic mechanism to regulate their growth rate relative to the local environment. According to this idea, heritable processes involving the perception of and reaction to space confinement may affect growth rate. The potential mediator(s) of this response to confined space are unknown. One obvious candidate, however, is sight: fish may be able to visually perceive the size of their surroundings, just as they do their social interactions, and regulate their growth rates accordingly. A related possibility is that cortisol, the most active and abundant corticosteroid in fish blood [7], may be involved in the conduction of an environmental cue. A key function of cortisol is in maintaining a balanced energy metabolism [8], which is crucial for determining growth rate. Furthermore, excess cortisol, released into the blood stream as a consequence of stress, is known to have detrimental effects on growth [9], immunological function [10], and sexual reproduction [11]. Consistent with this idea, aquaculture studies have discovered a positive correlation between stunted growth at high stocking densities and elevated plasma cortisol levels [4], [12], [13].

Here we examine the relationship between environmental space and growth in the Mexican tetra, *Astyanax mexicanus*, a single teleost species consisting of a sighted surface dwelling form (surface fish) and many different blind cave dwelling forms (cavefish) [14]. The surface and cave forms of *Astyanax* have evolved in contrasting spatial environments for the past few million years [15]. Surface fish are typically found in extensive bodies of fresh water, including large springs, rivers, and lakes, whereas cavefish inhabit more spatially restricted and seasonally affected pools in limestone caves. At least 29 separate cavefish populations, including some that evolved independently from surface fish ancestors [16], have been reported in northeastern Mexico [17]. In contrast to surface fish, which have large eyes and visually based behaviors, blind cavefish have degenerate eyes and compensate for vision by enhancing other sensory systems, such as the lateral line of neuromasts [18]–[20], taste receptors [21]–[23], and possibly the olfactory system [24], [25], and/or by evolving non-visually based behaviors [26]–[29]. Positive selection in the dark cave environment has resulted in the evolution of adaptive traits, such as vibration attraction behavior, in which cavefish respond to low frequency water vibrations by swimming toward their source [20], [30]. Because of the spatial limitations imposed by the cave environment, *Astyanax* cavefish provide an excellent model system to study the relationship between growth regulation and environmental space. Since surface fish and cavefish are inter-fertile [31], [32], the application of genetic approaches to test the inheritance of growth regulation can also be applied in the *Astyanax* system.

The present study has addressed several key questions concerning the relationship between environmental space and growth in *Astyanax mexicanus*. First, do *Astyanax* individuals respond to confined environmental space by adjusting their growth rate? Second, are there differences in the effects of confined space on growth in surface fish and cavefish, which have evolved in contrasting spatial habitats? Third, is vision required for the regulation of growth as a function of environmental space? Fourth, does SDG have a genetic basis? Lastly, is growth regulation under spatially confined conditions mediated by an elevation in plasma cortisol levels indicative of a stress response?

This study reveals that the teleost *Astyanax mexicanus* has an intrinsic mechanism to control growth as a response to environmental size, which we have termed space dependant growth (SDG). SDG is mediated by non-visual sensory receptors, has a genetic component, may be related to a stress response, and has regressed during the evolution of cavefish.

## Methods

### Ethics Statement

This study was carried out in strict accordance with the recommendations in the Guide for the Care and Use of Laboratory Animals of The National Institutes of Health. The protocol was approved by the Committee on the Ethics of Animal Experimentation of the University of Maryland (Permit R 09–58). All efforts were made to minimize suffering.

### Fish Rearing, Mating, and Care

Experiments were performed on laboratory-raised *Astyanax mexicanus* surface fish and cavefish (Pachón and Tinaja types), as well as F1 and F2 hybrids obtained from surface fish X Pachón crosses [33]. F1 hybrids were obtained by crossing a single male Pachón cavefish with three female surface fish. F2 hybrids were obtained by mating the F1 progeny of another surface fish x Pachón cavefish cross. Fish used in growth experiments were 2–10 months old, weighed 0.008–0.25 g, and ranged from 7–24 mm in length. Prior to use in a growth experiment, fish were maintained together in 2.5 l tanks, and fed brine shrimp daily. Water temperature was 18–20°C.

### Growth Experiments

Individual fish were selected at random from a tank population. Fish were padded dry, their length was measured from the tip of the lower jaw to the base of the spine, and they were weighed. This process took less than one minute, and the individual was then placed into a container with aquarium water. The same process was followed every time fish were measured and weighed during the experiments. As a precaution to prevent potential stress from brine shrimp, fish were only fed minimal amounts of brine shrimp the day after being measured.

Individual surface fish, Pachón cavefish, Tinaja cavefish, F1 hybrids, and F2 hybrids were raised for 18–36 days in 5, 10, or 40 ml volumes of water depending on the particular experiment. In some experiments, measurements of length and weight were done five times during this period, and in other experiments length and weight was recorded only at the beginning and end of the experiment. The rate of growth was determined by dividing the change in length and mass from the beginning to the end of the experiment by the number of days. Similar growth experiments were conducted on surface fish individuals grown in darkness.

Fish were fed equivalent amounts of live brine shrimp during the growth experiments. The amount of brine shrimp was determined by catering to the consumption rates of the larger fish to insure that fish were not food deprived. As a result, all fish were presented with an excess of food. On average, between 2–4 drops of fresh brine shrimp were fed to each fish per day.

### Growth Rate Under Static Conditions

**Figure 1:**
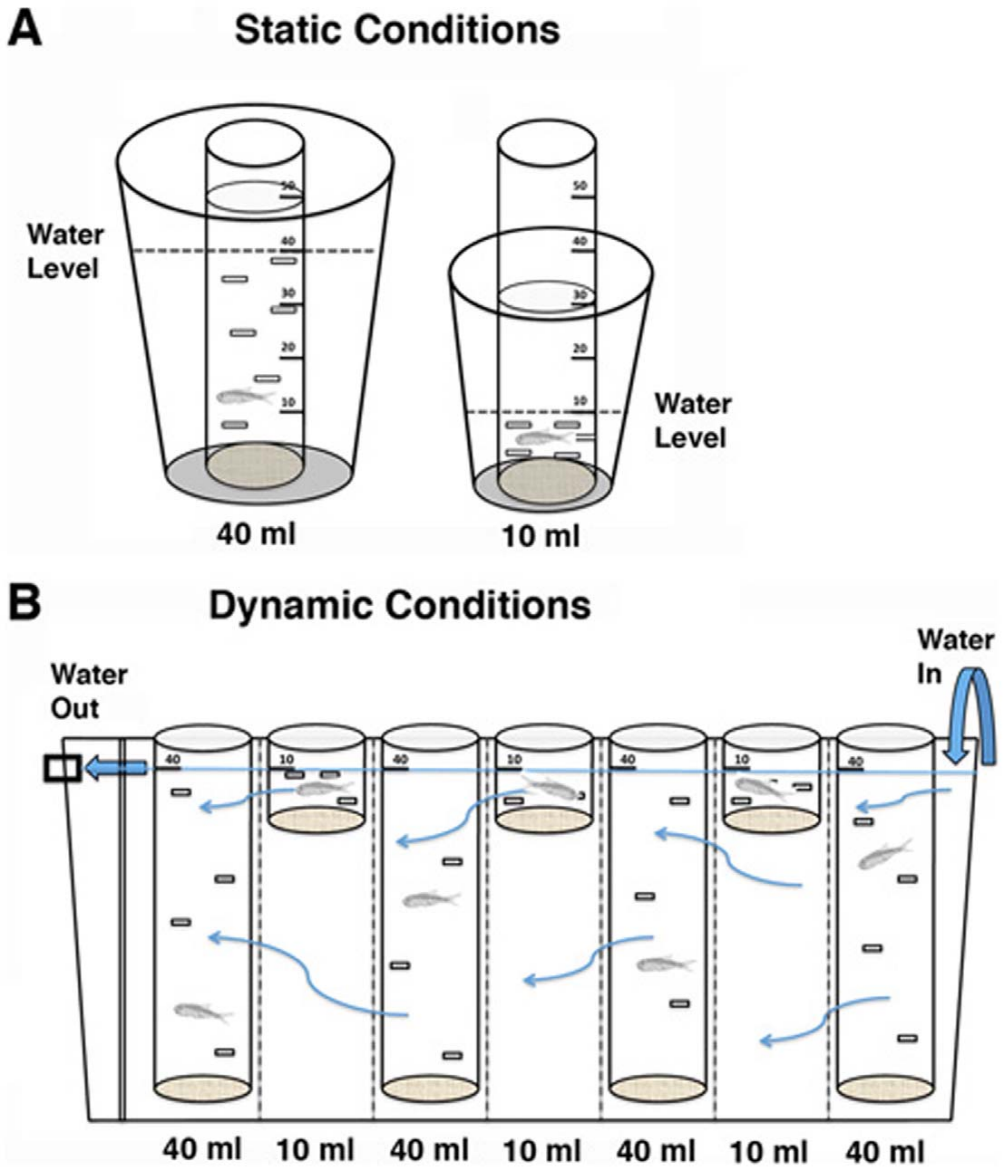
The apparatuses used to measure growth rates under static and dynamic conditions. Static conditions are depicted by panel A and dynamic, or flow, conditions are depicted by panel B.

The apparatus used to determine growth rate under static conditions is shown in Figure 1A. This apparatus allowed individual fish to swim freely in an inner chamber within a designated volume of water, and used an outer chamber of larger capacity as a water exchange reservoir. The inner containers for the 5 and 10 ml volumes (Fig. 1A, right) were constructed using 50 ml plastic conical tubes (VWR Scientific, Batavia, IL), which were inserted into 100 ml plastic beakers that served as the reservoir. To construct an inner container, 8–10 10 mm×2 mm slits were evenly distributed for the chosen volume and cut into each tube. The bottoms of the conical tubes were then cut off and replaced with a secured net consisting of a layer of cheesecloth. The inner chamber was placed into the reservoir with the cheesecloth bottom facing down, and water was added to the reservoir until it reached the appropriate volume in the inner chamber. The reservoir was covered with white paper to prevent fish from seeing outside the apparatus. A similar experimental design using 50 ml conical tubes as inner chambers was followed for the 40 ml volumes (Fig. 1A, left), but the reservoir was a 500 ml opaque plastic cup. Since the cups were already opaque, no covering was used on the outside.

A single fish was added to the water in each inner chamber. The water volume was kept constant by adding water daily to compensate for loss through evaporation, and complete water changes were made periodically during the course of the experiments. To account for differential water quality deterioration, water was changed based on the total volume of water present per condition. For the 5 ml, 10 ml, and 40 ml conditions water changes were performed every 2–3, 4, and 12 days respectively. For the water change, the inner container was lifted out of the outer container and put into another outer container with fresh aquarium water. This process took less than 10 seconds. To ensure that fish in the 5 and 10 ml conditions were not experiencing additional stress due to more frequent water changes, individuals in the 40 ml condition would undergo a “false” water change, where the inner container would be lifted out and then replaced into the same outer container. Dissolved oxygen, pH, nitrate, ammonia, and nitrite were also separately monitored for these conditions to confirm water quality.

### Growth Rate Under Dynamic Conditions

The apparatus used to determine growth rate under dynamic, or flow, conditions is shown in Figure 1B. The same 50 ml conical tubes as described above for the static growth conditions were used. Conical tubes were secured in a 1 l tank, with 7 conical tubes per tank. The tank functioned as a reservoir of continuously moving water: fresh water was constantly flowing into the tank at one end and draining from the tank at the opposite end. Conical tubes were secured to the sides of the tank so that the outside tank water level reached either the 10 ml or 40 ml gradation on the conical tube. The 10 ml and 40 ml conical tubes were alternated to account for slight differences in water exchange throughout the tank. Opaque plastic dividers with small holes throughout separated the conical tubes to prevent individual fish from seeing each other. Individual fish were then introduced, one per tube.

Fish were fed 2–4 drops of brine shrimp twice a day to account for the rapid sinking of brine shrimp through the cheesecloth netting at the bottom of the conical tube. Fish in the 10 ml and 40 ml volumes were fed equivalent amounts of food throughout the 27-day experimental period.

**Figure 2:**
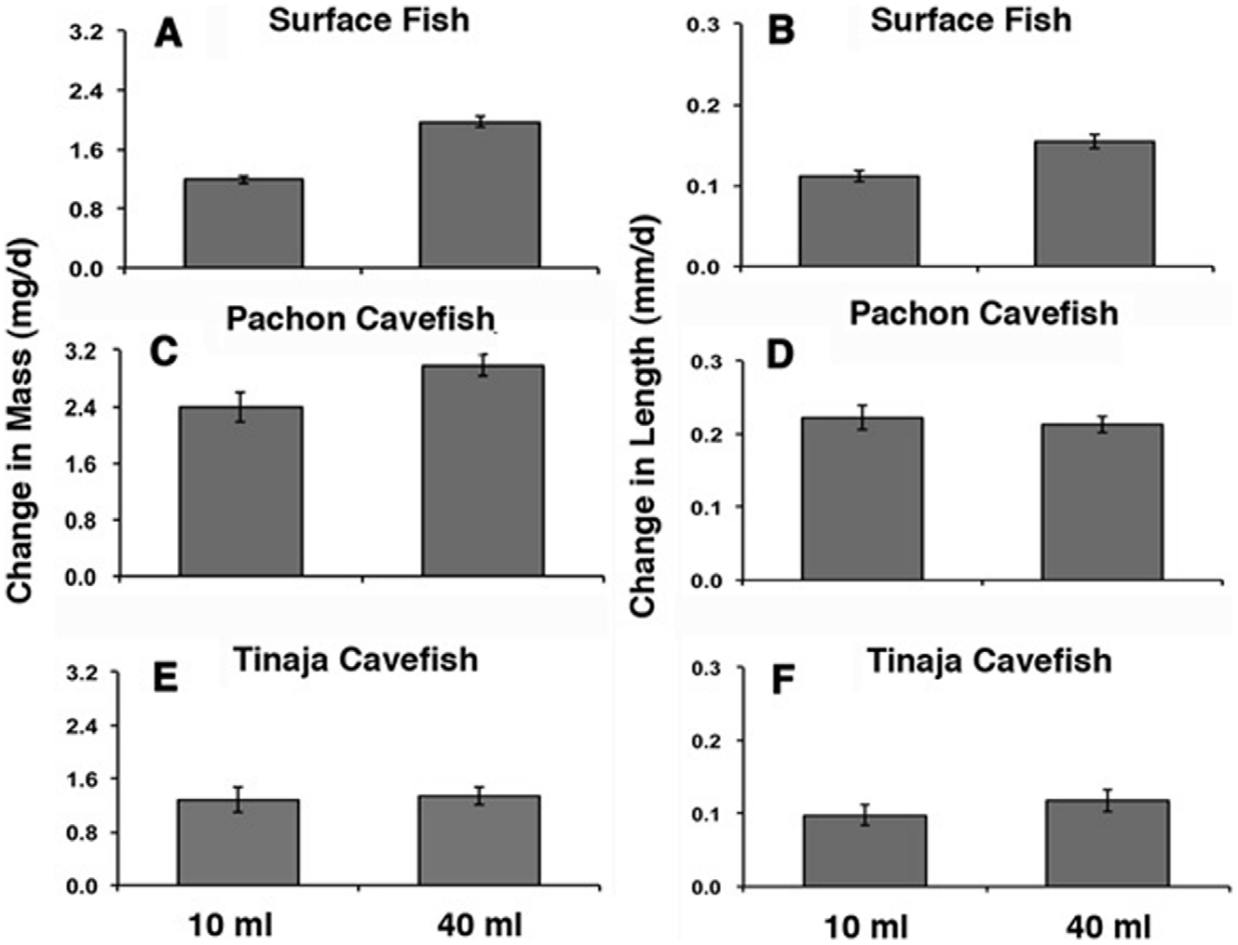
Space dependent growth under static conditions. The mean growth rate in terms of mass (A, C, E) and length (B, D, F) in surface fish (A, B), Pachón cavefish (C, D), and Tinaja cavefish (E, F) raised in 10 ml (left histograms in each frame) or 40 ml (right histograms in each frame) volumes for 32 days. Error bars: standard error of the mean. Asterisks indicate statistically significant differences in mean growth rate. See Table 1 for further information and statistical analyses.

### Growth Conditions for Cortisol Determination

For growth experiments involving cortisol analysis, 4 month-old individuals of about the same size and weight were selected and allowed to acclimate for a week in chambers containing 100 ml of water while being fed 3 drops brine shrimp daily. Following acclimation, control fish to be used for determining basal cortisol levels were euthanized with 500 mg/l Tricaine (Sigma-Aldrich, USA) and stored at −20°C for subsequent cortisol extraction. The remaining experimental fish were then raised under static conditions, as described above (Fig. 1A). At 1, 7, 14, 21, and 35 days during the growth experiments, fish were selected randomly, euthanized, and sacrificed at the same time each day to eliminate potential error based on normal cortisol fluctuations throughout the day [34], and stored at −20°C for subsequent cortisol extraction. Fish were not fed for 24 hours prior to sampling to reduce the possibility of elevated cortisol levels as a result of feeding [35].

### Cortisol Extraction and Analysis

A whole-body cortisol extraction protocol [36] was used to extract cortisol from individual fish. Fish stored at −20°C were allowed to thaw for 3–5 minutes prior to homogenization. Homogenization was performed with a 1 ml Wheaton glass homogenizer, which was rinsed twice with 100% ethanol and three times with distilled water between each sample to prevent cross-contamination. Ether extraction was performed twice for each sample. Following completion of the extraction protocol, samples were reconstituted with 1 ml of 1X PBS and kept at 4C until use. Extracted cortisol samples were used within one week.

Cortisol levels were analyzed using a human salivary cortisol assay kit (Salimetrics LLC, State College, PA) that had previously been found to give a positive assay with zebrafish [36]. The cortisol concentration of samples was determined by fitting a standard curve to a series of standards with known concentrations provided in the kit. Relative cortisol concentration per fish was calculated by dividing the determined cortisol concentration in the sample by the mass of the fish (g) prior to freezing. This gave a relative cortisol concentration of ng plasma cortisol/g fish mass.

**Figure 3:**
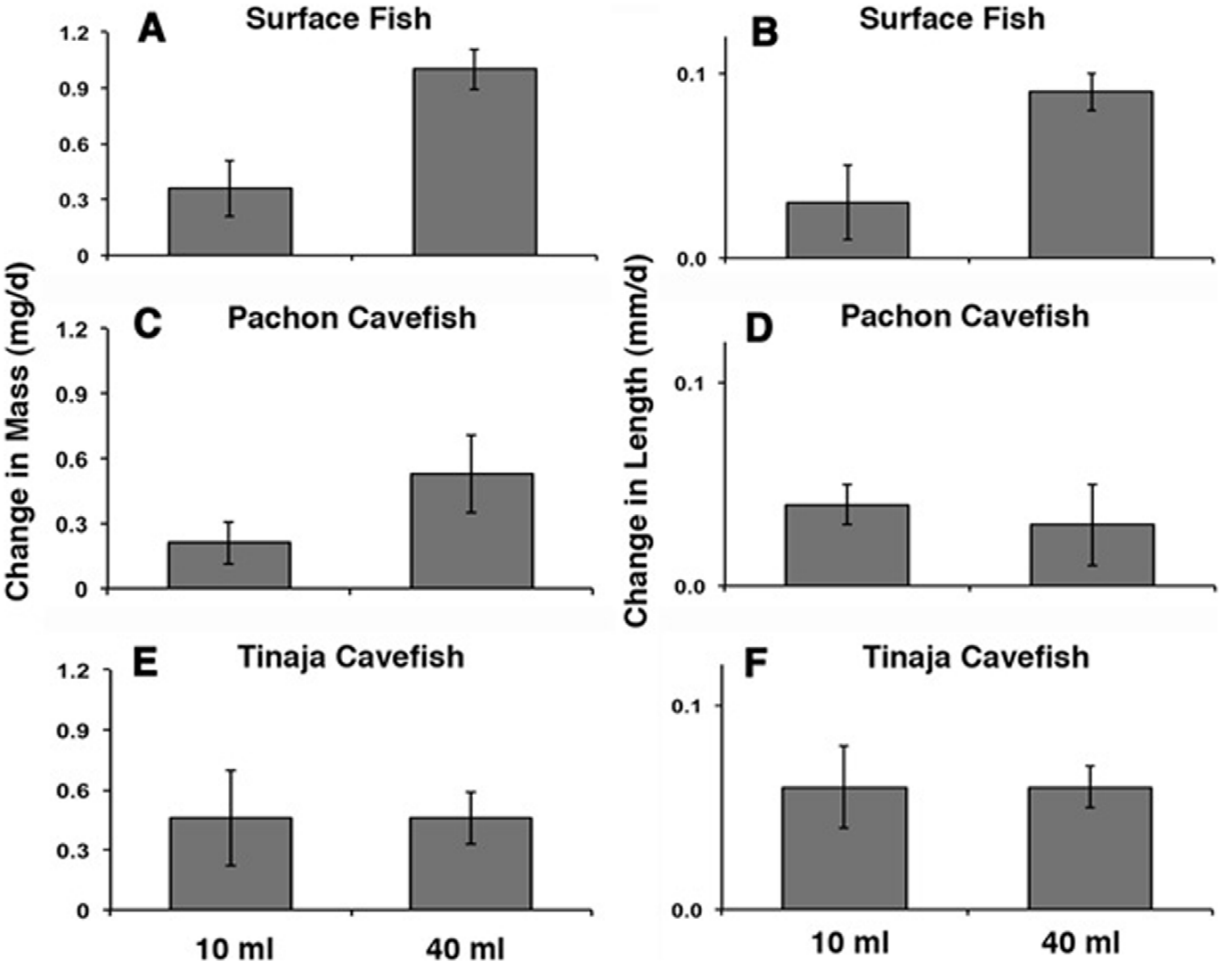
Space dependent growth under dynamic conditions. The mean growth rate in terms of mass (A, C, E) and length (B, D, F) in surface fish (A, B), Pachón cavefish (C, D), and Tinaja cavefish (E, F) raised in 10 ml (left histograms in each frame) or 40 ml (right histograms in each frame) volumes for 27 days. Error bars: standard error of the mean. Asterisks indicate statistically significant differences in mean growth rate. See Table 1 for further information and statistical analyses.

**Figure 4:**
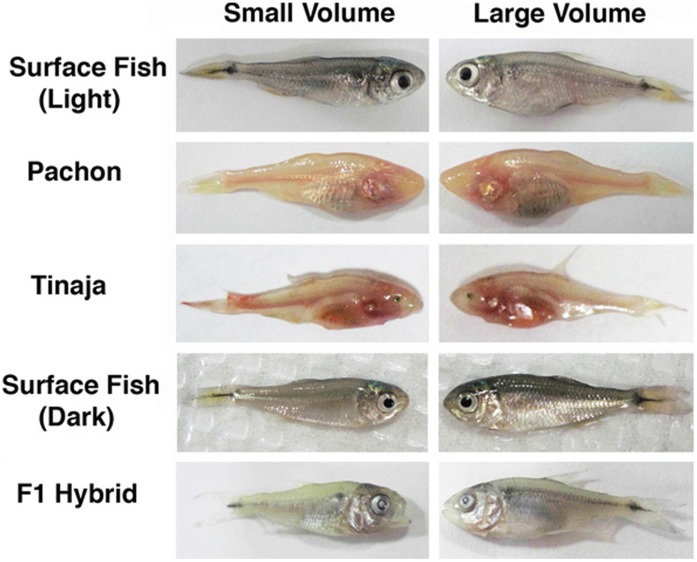
Differences between fish raised in small or large volumes of water under static conditions. Pairs of fish originally of the same initial weight and length were raised in 5 or 10 ml (left panels) or 40 ml (right panels) volumes in either light or darkness for 18–32 days. A, B. Surface fish raised in light. C, D. Pachón cavefish raised in light. E, F. Tinaja cavefish raised in light. G, H. Surface fish raised in darkness. I, J. F1 hybrids raised in light. Magnifications are the same in frames A and B, C and D, E and F, G and H, and I and J.

**Table 1:** Effects of confined space on growth rate in *Astyanax mexicanus* surface fish (SF), Pachón cavefish (CF), Tinaja cavefish (CF), and F1 and F2 hybrids.

					Mean Growth (+/− SE)		ANOVA (p)		Post Hoc Test^a^ (p)	
Subject	Conditions	Duration (days)	N	Volume (ml)	Mass (mg/d)	Length (mm/d)	Mass	Length	Mass	Length
SF	Static, Light	32	12	10	1.19+/−0.05	0.11+/−0.01	0.000	0.002	<0.01	<0.01
SF	Static, Light	32	12	40	1.97+/−0.07	0.15+/−0.01				
SF	Flow, Light	27	6	10	0.36+/−0.15	0.03+/−0.02	0.006	0.000	<0.01	>0.05
SF	Flow, Light	27	6	40	1.00+/−0.11	0.09+/−0.01				
CF Pachón	Static, Light	32	11	10	2.39+/−0.09	0.22+/−0.01	0.000	0.576	<0.01	–
CF Pachón	Static, Light	32	11	40	2.98+/−0.10	0.21+/−0.01				
CF Pachón	Flow, Light	27	5	10	0.21+/−0.10	0.04+/−0.01	0.186	0.705	–	–
CF Pachón	Flow, Light	27	6	40	0.53+/−0.18	0.03+/−0.02				
CF Tinaja	Static, Light	11	9	10	1.28+/−0.19	0.10+/−0.01	0.817	0.360	–	–
CF Tinaja	Static, Light	11	6	40	1.34+/−0.13	0.12+/−0.02				
CF Tinaja	Flow, Light	27	4	10	0.46+/−0.24	0.06+/−0.02	1.000	1.000	–	–
CF Tinaja	Flow, Light	27	4	40	0.46+/−0.13	0.06+/−0.01				
SF	Static, Dark	36	10	10	1.39+/−0.24	0.06+/−0.01	0.000	0.000	<0.01	<0.01
SF	Static, Dark	36	13	40	2.59+/−0.16	0.11+/−0.01				
F1 Hybrid	Static, Light	18	10	10	1.67+/−0.15	0.15+/−0.01	0.000	0.000	<0.01	<0.01
F1 Hybrid	Static, Light	18	9	40	4.44+/−0.11	0.29+/−0.02				
F2 Hybrid	Static, Light	32	10	10	2.19+/−0.11	0.19+/−0.02	0.005	0.260	<0.01	–
F2 Hybrid	Static, Light	32	12	40	2.66+/−0.09	0.21+/−0.01				

a Games-Howell post-hoc tests were used to analyze the data when Levene’s test was significant (p<0.05) and Tukey’s post hoc tests were used when Levene’s test was not significant (p>0.05).

### Statistics

PASW Statistics 18 software was used to analyze the data. Independent t-tests and one-way ANOVA were used to analyze the results of the growth experiments. When Levene’s test was significant (p<0.05), variance was assumed to be unequal, and Games-Howell post-hoc tests were used to analyze the data. When Levene’s test was not significant (p>0.05), variances were assumed to be equal, and Tukey’s post-hoc tests were used to analyze the data.

**Figure 5:**
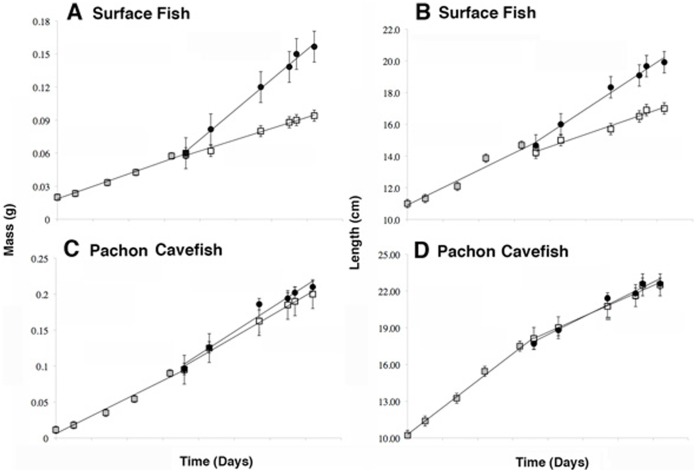
Stunted growth is reversible in surface fish. Individual fish were raised in confining volumes of water (10 ml) for 32 days, and then divided into two groups. One group was transferred to a larger volume of water (40 ml), the other was transferred to the same volume of water (10 ml), and both groups were raised in these conditions for an additional 40 days. A, B. Surface fish compensate for stunted growth in terms of mass (A) or length (B). C, D. Pachón cavefish show no appreciable change in growth rate in terms of mass (C) or length (D) when transferred from 10 ml to 40 ml volumes of water. Gray squares: growth rate in small volumes (10 ml). Black spheres: growth rate after fish raised in 10 ml volumes were transferred to 40 ml volumes. Open squares: growth rate after fish grown in 10 ml volumes were transferred into 10 ml volumes. Error bars represent standard errors of the mean.

## Results

### Space Dependent Growth in Surface Fish

To determine the effects of environmental space on growth rate, individual surface fish were raised in lighted chambers containing 10 ml or 40 ml of water for 27–32 days. Growth was compared under static conditions (Fig. 2), in which water was changed manually every few days, and dynamic conditions (Fig. 3), in which water flowed continuously throughout the experiments. Under static conditions, the mean growth rate of individuals maintained in the small volume was significantly lower in terms of both mass and length compared to individuals maintained in the large volume (Figs. 2A, B; 4A, B; Table 1). Similar results were obtained for growth under dynamic conditions: surface fish raised in the small volume had lower mean growth rates in terms of both mass and length compared to those maintained in the large volume (Fig. 3A, B; Table 1). The results indicate that surface fish growth is constrained by environmental space when measured under static or dynamic conditions. We have termed this space dependent growth (SDG), which is defined as a significant change in growth rate in response to environmental size.

We conducted additional experiments to determine whether the reduced growth rate exhibited by surface fish in confined space is reversible. In these experiments, individual fish raised in 10 ml volumes for 32 days and exhibiting SDG were transferred to 40 ml volumes and their growth rate was determined for an additional 40 days under static conditions. When compared to controls transferred to the same confined volume, the fish transferred to larger volumes showed an increased growth rate by mass (Fig. 5A) and length (Fig. 5B), which was even higher than fish raised continuously in unconfined conditions. The results indicate that surface fish can compensate for lost growth following a period of confinement by enhancing their growth rate in response to a larger environmental size.

### Regression of Space Dependent Growth in Cavefish

Similar experiments to those described above were carried out to determine the status of SDG in Pachón and Tinaja cavefish. Under static conditions, the growth rate of Pachón cavefish was decreased significantly by mass during maintenance in small compared to large volumes (Figs. 2C; 4C, D; Table 1), indicating that they exhibit SDG. However, the difference in growth rates measured by mass was not as pronounced as that seen in surface fish maintained under the same conditions, suggesting that the extent of SDG in terms of mass is reduced in Pachón cavefish. In contrast, no significant differences in growth rate determined by length were observed in Pachón cavefish grown in small or large volumes (Figs. 2D; 4C, D; Table 1), showing that SDG is absent according to this criterion. In the dynamic conditions, Pachón cavefish raised in small or large volumes of water showed no significant differences in growth rate in terms of mass and length (Fig. 3C, D; Table 1). Growth rate by mass was increased in the larger volume compared to the small volume, however this difference was not statistically significant (Fig. 3C; Table 1). Furthermore, Pachón cavefish raised in small volumes did not markedly increase their growth rate after transfer to larger volumes (Fig. 5C, D). Tinaja cavefish raised in small or large volumes failed to show significant reductions in growth rate when compared by mass or length under either static or dynamic conditions (Figs. 2E, F; 3E, F; 4E, F; Table 1). Therefore, SDG appears to be absent in Tinaja cavefish when measured by either growth criterion.

In summary, the results show that SDG has regressed to different extents in two types of cavefish. Furthermore, the results indicate that growth by mass can be uncoupled from growth by length in Pachón cavefish.

### Space Dependent Growth Does Not Require Vision

**Figure 6:**
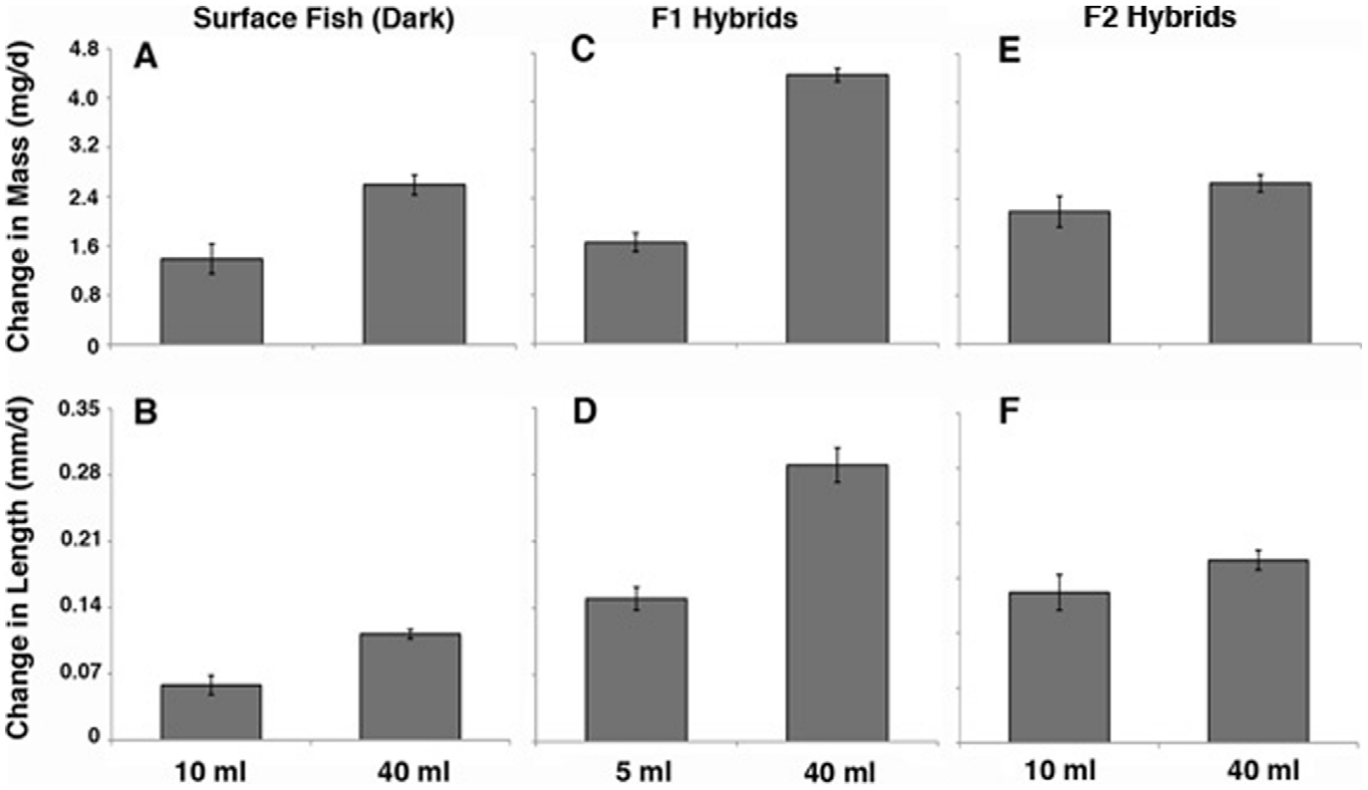
Space dependent growth does not require vision and has a genetic basis. A, B. Surface fish grown in darkness show SDG. C, D. F1 hybrids show SDG. E, F. F2 hybrids do not exhibit SDG in terms of length, but do exhibit limited SDG in terms of mass. Each frame shows the mean growth rate in terms of mass (A, C, E) and length (B, D, F). Other details are the same as in Figure 2.

The regression of SDG in cavefish, which lack functional eyes, opens the possibility that SDG may be mediated by vision. Since the experiments described above were conducted in light, surface fish were able to visualize the size of their container and could have adjusted their growth accordingly. In contrast, cavefish would not have been able to adjust their growth based on vision. To determine whether vision is required for SDG, we examined the growth rate of surface fish raised in different volumes for 36 days in darkness. Since previous studies showed there were no differences in the results obtained under static or dynamic conditions (Figs. 2, 3; Table 1), we used static conditions in these and all subsequent experiments. The levels of SDG in surface fish raised in darkness did not differ significantly from those raised in light (Figs. 4G, H; 6A, B; Table 1). On the basis of these findings, we conclude that vision is not required for SDG.

### Space Dependent Growth is a Dominant Genetic Trait

To investigate the inheritance of SDG, the mean growth rates of surface fish X Pachón cavefish hybrids raised in small or large volumes of water were compared as described above. The results of experiments with F1 hybrids revealed SDG at levels even more pronounced than those detected in surface fish in terms of mass and length (Fig. 4I, J; 6C, D; Table 1). The results demonstrate that SDG has a genetic basis and is inherited as a dominant trait.

In contrast to F1 hybrids, F2 hybrids showed an intermediate SDG phenotype with significant SDG in terms of mass (Fig. 6E; Table 1), but no significant SDG in terms of length (Fig. 6F; Table 1). However, inspection of individual F2 hybrids indicated that there was a great deal of variation and decoupling in the SDG phenotype. Some individuals showed SDG in terms of mass but not in terms of length, whereas others showed SDG in terms of length but not in terms of mass, indicating that SDG segregated in the F2 generation. The results imply that SDG is a multigenic trait.

### Cortisol Induced Stress and Space Dependent Growth

**Figure 7:**
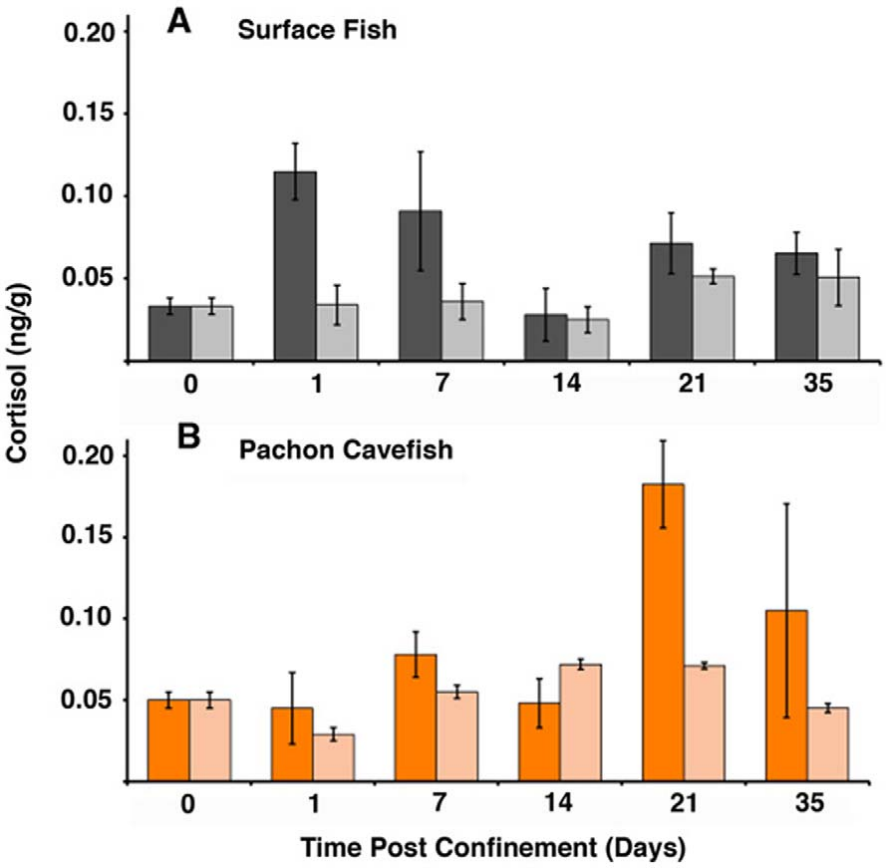
Surface fish and cavefish show a differential plasma cortisol response to confinement. Cortisol levels in surface fish (A) and Pachón cavefish (B) raised in confined (5/10 ml; left bar for each day) or unconfined (40 ml; right bar for each day) conditions for 35 days. Errors bars represent standard error of the mean. Asterisks indicate statistically significant cortisol levels. See Table 2 for further information, basal cortisol values for controls raised in 100 volumes, and statistical analyses.

To test the possibility that stress is involved in SDG, we compared cortisol levels in individual surface fish and Pachón cavefish raised in small or large volumes of water. Cortisol measurements were done at five time points over a five-week span and compared to basal levels in controls. Surface fish maintained in small volumes showed a 3–4 fold increase in cortisol when measured a day after the beginning of confinement relative to counterparts maintained in large volumes and controls (Fig. 7A; Table 2). Cortisol levels were subsequently reduced and gradually returned to normal after the first two weeks of confinement (Fig. 7A; Table 2). Pachón cavefish showed a different cortisol profile when subjected to the same confinement conditions as surface fish (Fig. 7B; Table 2). In contrast to surface fish, no significant elevation in cortisol was detected during the first 2–3 weeks following confinement, but a delayed increase was observed at 3-weeks post confinement. Afterwards, cortisol levels returned to normal. The results imply that confined space induces an immediate stress response in surface fish, which is substantially delayed in Pachón cavefish.

**Table 2:** Effects of confined space on cortisol levels in *Astyanax mexicanus* surface fish (SF) and Pachón cavefish (CF).

Subject	Conditions	Duration(days)	N	Volume(ml)	Plasma Cortisol(ng/g) (+/− E)	Significance(p)	Significance (p<0.05)
SF	Control	0	11	100	0.033+/−0.005	0.031	**
CF Pachón	Control	0	12	100	0.050+/−0.005		
SF	Confined Growth	1	3	5/10	0.115+/−0.017	0.017	**
SF	Normal Growth	1	3	40	0.034+/−0.012		
SF	Confined Growth	7	5	5/10	0.091+/−0.036	0.180	
SF	Normal Growth	7	5	40	0.036+/−0.011		
SF	Confined Growth	14	3	5/10	0.028+/−0.016	0.878	
SF	Normal Growth	14	3	40	0.025+/−0.008		
SF	Confined Growth	21	2	5/10	0.071+/−0.018	0.274	
SF	Normal Growth	21	2	40	0.051+/−0.005		
SF	Confined Growth	35	2	5/10	0.065+/−0.012	0.453	
SF	Normal Growth	35	3	40	0.050+/−0.017		
CF Pachón	Confined Growth	1	3	5/10	0.045+/−0.022	0.525	
CF Pachón	Normal Growth	1	3	40	0.029+/−0.004		
CF Pachón	Confined Growth	7	5	5/10	0.078+/−0.014	0.147	
CF Pachón	Normal Growth	7	5	40	0.055+/−0.004		
CF Pachón	Confined Growth	14	3	5/10	0.048+/−0.015	0.182	
CF Pachón	Normal Growth	14	3	40	0.072+/−0.003		
CF Pachón	Confined Growth	21	2	5/10	0.183+/−0.027	0.028	**
CF Pachón	Normal Growth	21	2	40	0.071+/−0.002		
CF Pachón	Confined Growth	35	2	5/10	0.105+/−0.066	0.328	
CF Pachón	Normal Growth	35	2	40	0.045+/−0.003		

## Discussion

The major finding of the present investigation is that the surface dwelling form of *Astyanax mexicanus* has an intrinsic capability to regulate its growth rate according to environmental space, which was termed SDG. It was also discovered that blind cavefish raised in different sized environments under the same conditions either lack or exhibit a reduction in SDG. In Tinaja cavefish, SDG is absent when measured by mass or length. In Pachón cavefish, SDG is detectible by mass, although at lower levels than in surface fish, but it is not detected when measured by length, indicating that the two growth parameters can be uncoupled during evolution. Along with the loss of eyes and pigmentation [26], [28], [29], SDG is an example of a regressive phenotype that has appeared during cavefish evolution.

### Space Dependent Growth

Previous studies showed that teleosts raised in higher stocking densities grew more slowly and eventually became stunted compared to those maintained in lower stocking densities [1]–[4]. However, these results were insufficient to conclude whether or not there is an intrinsic growth regulating process in these species because populations, rather than individuals, were studied, and it is known that social interactions between fish can affect their growth [5], [6]. In contrast, here we report growth regulation as a function of environmental size in individual fish and thus are able to rule out the effects of group interactions. Furthermore, the previous studies suggested that stunted growth of fish grown in confined environments was due to effects of water quality rather than the ability to intrinsically control growth rate according to space. Our studies addressed this issue by comparing growth in confinement, under conditions in which water was frequently manually changed, as well as in a flow through system in which water was continuously exchanged during the course of the experiments. Since no differences were observed in the effects of environmental space on growth in the two conditions, we were able to conclude that *Astyanax* surface fish have the capacity to regulate growth according to local environmental size, and not due to extrinsic water quality factors. To our knowledge, this is the first demonstration of an intrinsic process that regulates growth based on environmental size in teleosts.

In addition to being able to downregulate their growth rate in confined conditions, surface fish can compensate for lost growth by increasing their growth rate to high levels after being transferred from confined to unconfined environmental spaces. This remarkable plasticity in growth allows an individual to quickly recover from stunting, increase their size, and remain competitive in the population. Similar compensatory growth has been reported in individual teleosts exposed to a period of poor nutrition [37], [38].

### Sensory Receptors for Space Dependent Growth

The response of growth to confined environment space is likely to occur in two steps: first, confined space is perceived by a sensory system, and second, growth parameters are modulated according to the received inputs. The regression of SDG in cavefish, which live in complete darkness and lack functional eyes, initially suggested that confined environmental space may be perceived by vision in surface fish. However, we have found that SDG also occurs when surface fish are raised in the dark. Thus our results demonstrate that the effects of confined space on growth are not manifest through sight but must instead involve non-visual sensory receptors.

The non-visual sensory receptors by which cavefish (and surface fish) perceive environmental space remain to be identified. However, various types of non-visual sensory organs, such as the lateral line of neuromasts, are obvious candidates. The lateral line system detects water movements in *Astyanax* [19], [20]. The neuromasts and their underlying neural circuitry could be used to perceive space by processing water movements reflected from the boundaries of the aqueous environment as fish swim through it. If the lateral line sensory system is involved in mediating SDG, however, then the underlying changes must be qualitative rather than quantitative because neuromasts are increased in size and number in cavefish compared to surface fish [18], [20]. A possibility for future consideration is that the regression of SDG in cavefish involves rewiring of the lateral line sensory circuitry, perhaps due to positive selection for neuromast-mediated vibration attraction behavior [20], and/or fundamental modifications in the responsive centers in the brain.

### Inheritance and Uncoupling of Space Dependent Growth

The growth experiment with F1 hybrids revealed that SDG has a large genetic component and is inherited as a dominant trait. Dominant inheritance of SDG may explain why this trait has not been entirely lost when measured by mass in Pachón cavefish. Cavefish from the Pachón Cave are thought to have experienced a recent hybridization event with surface fish from the surrounding area, in which dominant SDG genes could have been introduced into the cave population [39]–[41]. There is no evidence for such a hybridization event in Tinaja Cave. We conclude that SDG has a genetic component, providing further support for the possibility that fish regulate their growth through an intrinsic process mediated by sensory cues.

In F2 hybrids, some individuals showed SDG in terms of mass but not in terms of length, while others showed SDG in terms of length but not in terms of mass. Consistent with this expectation, there was significant variation in the intensity of SDG expression in the F2 generation, showing the potential for intermediate phenotypes. These results suggest that there are multiple genetically controlled components of SDG that are independently segregated during meiosis. The control of growth requires a complex set of interactions that ultimately determine the size of an organism and thus it is not unexpected that SDG is likely to be controlled by multiple genes in *Astyanax*.

Several lines of evidence suggest that the underlying processes involving SDG by mass and length are independent. First, the effects on growth by confined space were greater when measured by mass than by length in Pachón cavefish. Second, SDG measured in terms of mass and length segregate independently in F2 hybrids. Growth rate in terms of mass is likely related to the appetite of individuals as well as metabolic processes, while growth rate in terms of length is probably related to body axis extension, which includes muscle, cartilage, and bone formation. While both of these processes are affected by environmental space, their underlying molecular and physiological mechanisms are probably different.

### Is Regression of Space Dependent Growth Adaptive in Cavefish?

In contrast to surface fish, which are usually found in large bodies of water, cavefish inhabit relatively small cave pools, at least during the seasons in which floodwaters do not sweep through caves. Does SDG regress by drift in cavefish because there is no selective advantage for this trait in relatively confined pools or because it is in some way adaptive not to restrict growth under these conditions? We cannot answer this question with certainty in the absence of knowledge about growth rates in the natural cave environment, but it can be speculated that the regression of SDG may be adaptive in cavefish under the conditions of seasonal fluctuations in food availability. Caves are habitats without primary productivity. The food sources available for *Astyanax* cavefish are deposited in caves by the daily activity of roosting bats and by seasonal flooding [17]. The activity of bat colonies seems to vary among different caves, but almost all of them would be subject to the effects of seasonal flooding. Flooding could produce spikes of food input from the surface environment, and competition for this unpredictable resource could be strong between cavefish. Accordingly, it may be adaptive for cavefish to grow to large size as quickly as possible irrespective of environmental space to compete with their con-specifics for this limited resource. SDG could limit the fitness of cavefish under these conditions and thus be subject to strong negative selection. This explanation for regression of SDG is similar to that recently proposed to account for reduced sleep activity in cavefish, another regressive trait that may have evolved to counteract sporadic food availability [42].

Future studies should address additional factors that may be related to the adaptive significance of SDG regression in cavefish, such as cave pool water quality, frequency of surface fish invasions (in caves subjected to hybridization), and the density of cavefish populations, as well as expand the analysis of SDG to other *Astyanax* cave populations to determine if any particular environmental factor(s) leads to the suppression of this trait.

### Role of Stress in Space Dependent Growth

The present study has shown that levels of cortisol are substantially elevated within a day after surface fish are confined to a small environmental space. This finding, along with absence of a corresponding cortisol increase in Pachón cavefish, suggests that the initial trigger for SDG could be a stress response. It is unlikely, however, that sustained effects on growth are stress related because cortisol levels in surface fish gradually decrease to normal in confined conditions. Furthermore, SDG was reduced or absent in Pachón cavefish despite a delayed increase in cortisol levels. Therefore, secretion of cortisol into the plasma may not be the single factor driving the presence or absence of SDG, although it could be a determinant responsible for initiating growth plasticity in surface fish. The hypothalamus initiates the first step of the stress response in teleosts [43]. Accordingly, it is interesting to note that cavefish have modified the developmental processes related to hypothalamus formation [25]. Therefore, evolutionary changes in the hypothalamus could be responsible for suppression of the initial stress response and SDG regression in cavefish.

### Conclusions

This study has filled major gaps in our understanding of how teleosts regulate their growth in a constrained spatial environment. First, we have shown that individual *Astyanax* surface fish can determine the size of their environment and regulate their growth rate accordingly. Decreased growth in limiting spatial conditions was not due to external factors, such as food or water quality, but was instead due to the direct downregulation of growth rate mediated by sensory cues. Second, we have shown that different populations of cavefish have regressed SDG to different degrees, perhaps as an adaptive response to their naturally constrained environments. Third, we have demonstrated that vision is not necessary for SDG, suggesting that other sensory systems must be used to determine the size of the local environment. Fourth, this study suggests that a cortisol-based stress response may operate in determining growth rates in response to confinement, but does not solely determine the presence or absence of SDG. Lastly, we have shown that SDG has a largely genetic basis. This study opens the possibility of future research that could provide more insight into a novel phenotype that allows teleosts to exhibit remarkable growth plasticity in response to the size of their environment.
